# Cervical Dysplasia, Infection, and Phylogeny of Human Papillomavirus in HIV-Infected and HIV-Uninfected Women at a Reproductive Health Clinic in Nairobi, Kenya

**DOI:** 10.1155/2020/4945608

**Published:** 2020-06-16

**Authors:** Agnes Omire, Nancy L. M. Budambula, Leah Kirumbi, Hillary Langat, Danvas Kerosi, Washingtone Ochieng, Raphael Lwembe

**Affiliations:** ^1^Jomo Kenyatta University of Agriculture and Technology, P.O. Box 62000-00200, Nairobi, Kenya; ^2^University of Embu, P.O. Box 6-60100, Embu, Kenya; ^3^Kenya Medical Research Institute, P.O. Box 54840-00100, Nairobi, Kenya; ^4^Center for Virus Research in Therapeutic Sciences, P.O. Box 59857-00200, Nairobi, Kenya

## Abstract

High risk human Papillomavirus (HPV) infections ultimately cause cervical cancer. Human Immunodeficiency Virus (HIV) infected women often present with multiple high-risk HPV infections and are thus at a higher risk of developing cervical cancer. However, information on the circulating high-risk HPV genotypes in Kenya in both HIV-infected and HIV-uninfected women is still scanty. This study is aimed at determining the phylogeny and the HPV genotypes in women with respect to their HIV status and at correlating this with cytology results. This study was carried out among women attending the Reproductive Health Clinic at Kenyatta National Hospital, a referral hospital in Nairobi, Kenya. A cross-sectional study recruited a total of 217 women aged 18 to 50 years. Paired blood and cervical samples were obtained from consenting participants. Blood was used for serological HIV screening while cervical smears were used for cytology followed by HPV DNA extraction, HPV DNA PCR amplification, and phylogenetic analysis. Out of 217 participants, 29 (13.4%) were HIV seropositive, while 68 (31.3%) were positive for HPV DNA. Eight (3.7%) of the participants had abnormal cervical cytology. High-risk HPV 16 was the most prevalent followed by HPV 81, 73, 35, and 52. One participant had cervical cancer, was HIV infected, and had multiple high-risk infections with HPV 26, 35, and 58. HPV 16, 6, and 81 had two variants each. HPV 16 in this study clustered with HPV from Iran and Africa. This study shows the circulation of other HPV 35, 52, 73, 81, 31, 51, 45, 58, and 26 in the Kenyan population that play important roles in cancer etiology but are not included in the HPV vaccine. Data from this study could inform vaccination strategies. Additionally, this data will be useful in future epidemiological studies of HPV in Nairobi as the introduction or development of new variants can be detected.

## 1. Introduction

Human Papillomaviruses (HPVs) are divided into three groups [1]. Group 1 HPV is known to be carcinogenic and includes HPV 16, 18, 31, 33, 35, 45, 52, and 58. Group 2 includes those that are probably carcinogenic and includes HPV 68, 53, 66, 67, 70, 73, and 82 while group 3 includes those with no carcinogenic ability (HPV 6 and 11). The most prevalent HPV genotypes globally in a decreasing order are HPV 16, 18, 33, 45, 31, 58, 52, 35, 59, 56, 51, 39, 6, 68, 73, 66, and 70 [2]. HPV infection is the main risk factor for cervical cancer which is the leading cause of mortality in Africa [3, 4].

Compared to HIV-uninfected women, HIV-infected women are at an increased risk for HPV infection and tend to be infected with multiple genotypes of HPV [5–7]. HIV-infected women are also more likely to be infected with high-risk HPV genotypes that are not in the HPV vaccines [8]. The most oncogenic HPV is the alpha 9 (16, 31, 35, 58, 67, and 52) and alpha 7 (59, 18, 45, 70, 39, 68, and 85) papillomaviruses [9]. Of these, HPV 16 and 18 are the most prevalent oncogenic types in cervical malignancies causing up to 70% of all malignancies [2, 10]. It is for these reasons that they are included in the HPV vaccines. There are three main vaccines for cervical cancer: a bivalent vaccine (Cervarix) that contains HPV 16 and 18 only, a quadrivalent vaccine (Gardasil) that has HPV 6, 11, 16, and 18, and a 9 valent vaccine with HPV 16, 18, 11, 6, 31, 33, 45, 52, and 58 [11, 12]. These vaccines are a breakthrough for cancer prevention since they have the HPV genotypes that are most oncogenic [13, 14]. However, other high-risk types seem to be playing important roles in cervical cancer etiology. In addition, the distribution of high-risk HPV has been shown to vary geographically [15].

A sequence of the L1 region of HPV is used for taxonomic analysis where more than 10%, 2-10% and less than 2% in sequence difference would represent different types, subtypes, and variants, respectively [16]. Phylogenetic analysis is important for the analysis of viral genes with high rates of mutation and for determining the patterns of viral emergence [17]. HPV variant analysis is important for precise diagnosis and vaccine design [18]. This study is therefore aimed at determining the HPV genotypes found in women with respect to their HIV status and their phylogenetic relationship and at correlating the genotypes with cytological endpoints which reflect cervical cancer or the progression towards cervical cancer.

## 2. Materials and Methods

### 2.1. Study Site and Ethical Approval

A cross-sectional study was done at the National Reproductive Health Clinic at Kenyatta National Hospital, a referral hospital in Nairobi, Kenya. This was after study approval by the National Ethical Review Committees at the Kenya Medical Research Institute and Kenyatta National Hospital. The Reproductive Health Clinic at Kenyatta National Hospital is a referral center for cervical cancer screening, family planning services, and fistula care.

### 2.2. Study Participants

Recruitment of participants was done as they walked into the KNH Reproductive Health Clinic between August 2013 and April 2014. A minimum sample size was arrived at by using a HPV prevalence of 27% [7]. The minimum sample size was calculated as 215 [19]. However, 217 individuals were recruited in the study. Participants were women between the ages of 18 and 50 years who consented to the study. Those who did not consent and those of ages below 18 years or above 50 years were excluded from the study. This is because above 50 years, it is not possible to reach the squamocolumnar junction since it is taken up into the endocervical canal in postmenopausal women [20]. This cut off of 50 years was arrived at based on a study done on Kenyan women that reported the age of menopause to be 48.28 years [21]. Pregnant women were also excluded because of fear of loss of the pregnancy and because many women experience some bleeding after a vaginal swab that could cause anxiety.

### 2.3. Demographic Information

A questionnaire was used to collect demographic information including age of the participants, marital status, level of education, and parity.

### 2.4. Biological Specimen Collection for HIV Testing

All consenting participants were given pre- and post-HIV counseling and tested for HIV infection using the Kenya National HIV Testing Algorithm [22]. Determine™ HIV rapid test was used as the first rapid test for all participants; all cases reactive to Determine™ were screened again using the second rapid test UniGold™. In case of indeterminate results, 3 ml of blood was collected for an ELISA test to confirm the HIV status at the Kenya Medical Research Institute [23].

### 2.5. Cervical Swab Collection for HPV Testing

The patient was requested to get on the couch and lie on their back in a lithotomy position. A speculum examination was done, and the cervical os (opening of the cervix) was visualized. Cervical smears were then collected by a nurse by inserting a cytology brush into the endocervical canal making sure ectocervical and endocervical cells are taken. The brush was rotated one full turn and then removed gently. The cervical epithelial cells obtained were spread on a microscopic slide for cytology, after which the brush was immediately rinsed in vials containing virus transport media and the brush detached into the media. The vials were tightly closed and stored at -20°C awaiting viral DNA extraction, PCR amplification, and phylogenetic studies.

### 2.6. Cytology

The cervical smears were analyzed under the strict supervision of a cytotechnologist who was supervised by a resident pathologist at the Kenya Medical Research Institute. An independent pathologist and a second cytotechnologist were involved to ensure the quality of cytology results. The patients were called back for repeat pap smear if the sample was unsatisfactory due to blurring by blood, presence of a lot of pus cells, or insufficient cells to allow adequate assessment [20]. Fixed smears were stained to enable visualization of the cell nucleus and cytoplasm. Changes in the size of the nucleus, presence of koilocytosis, or changes in the nuclear to cytoplasm ratio were sought after the staining. Cervical cytology results were reported according to the Bethesda system as negative for intraepithelial lesion or malignancy, Atypical Squamous Cells of Undetermined Significance (ASCUS), atypical squamous cells-cannot exclude high-grade squamous intraepithelial lesion (ASC-H), Low-grade Squamous Intraepithelial Lesions (LSIL), High-grade Squamous Intraepithelial Lesions (HSIL), squamous cell carcinoma, endocervical adenocarcinoma in situ, and adenocarcinoma [24].

### 2.7. HPV DNA Extraction and Amplification

Vials containing the cytology brush were thawed and vortexed to release the material from the brush into the medium. HPV DNA extraction was done for all the samples using the QIAamp DNA kit (Qiagen, Germany) following the blood and body fluid spin protocol. The DNA was eluted in 50 *μ*l of RNAse and DNAse free water. PCR amplification of the HPV LI region was done using HPV consensus primers that target a 450 base pair region in the L1 open reading frame of HPV using the MY09/MY11 primers and then reamplified using the nested GP5+/GP6+ primers that amplify a 150 bp region [23]. L1 genome is highly conserved between HPV genotypes and hence used for taxonomic analysis. In the primary PCR, 5 *μ*l of the extracted product, 2.0 mM MgCl_2_, 500 nM MY09, 500 nM MY11, and 400 *μ*M of dNTPs were used. The thermocycler programming was set as follows: initial activation at 95°C for 10 minutes followed by 40 cycles of denaturation at 95°C for 30 seconds, annealing at 48°C for 30 seconds, and extension at 72°C for 30 seconds [23]. The final extension was at 72°C for 10 minutes. In the nested PCR, 5 *μ*l of the primary PCR product, 2.0 mM MgCl_2_, 500 nM of GP5+, 500 nM of GP6+, and 400 *μ*M of dNTPs were used. The thermocycler programming was set as follows: initial activation at 94°C for 10 minutes followed by 40 cycles of denaturation at 94°C for 30 seconds, annealing at 43°C for 30 seconds, and extension at 72°C for 30 seconds. The final extension was at 72°C for 10 minutes. Amplitaq Gold Applied Biosystems was used in each PCR run. DNAse free RNAse free water was used as the negative control in each PCR run.

Electrophoresis was done using a 2% agarose gel with 5 *μ*l aliquot of the PCR product. Samples showing a band of approximately 140 bp were confirmed as positive for HPV [7, 23]. The HPV-positive samples after nested PCR were then purified and sequenced. Purification of the PCR-positive products was done using the QIAquick DNA purification kit (Qiagen, Germany) and sequenced using the BigDye Terminator (Life Technologies).

### 2.8. Data Analysis

Demographic data was analyzed using SPSS software version 18.0. A Chi-square test was used for comparison of categorical data. Fisher's exact test was used for data with a frequency of less than 5. Continuous data was represented in terms of means and standard deviation. Statistical significance was set when *p* value was less than 0.05.

### 2.9. HPV Typing and Phylogenetic Analysis

The phylogenetic tree was constructed using the sequences of the L1 region. Partial sequences of the amplified HPV L1 region were edited in Chromas software Version 2.4.3. The sequences were then blasted on NCBI http://blast.ncbi.nlm.gov/blast.cgi. Representative sequences of the same type with unique differences were selected for phylogenetic analysis [18]. Reference sequences were retrieved from Genbank. An input file was then prepared with all the representative sequences and reference sequences. These sequences were used for multiple sequence alignment with CLUSTAL W in MEGA X software [25]. The evolutionary history was inferred by using the Maximum Likelihood method and the Tamura-Nei model [26]. Initial trees for the heuristic search were obtained automatically by applying Neighbor-Join and BioNJ algorithms to a matrix of pairwise distances estimated using the Maximum Composite Likelihood (MCL) approach and then selecting the topology with superior log likelihood value. This analysis involved 61 nucleotide sequences. Codon positions included were 1st+2nd+3rd+noncoding. There was a total of 155 positions in the final dataset. Branches with bootstrap replicates less than 50% were collapsed. The tree was drawn to scale, with branch lengths measured in the number of substitutions per site. TreeView was used to visualize the tree [27].

## 3. Results

### 3.1. Demographic Information

A total of 217 women were recruited into the study with a median age of 36 years, mean age of 35.73 years, and standard deviation of 7.4. Over half of the women (60.8%) were between 25 and 39 years while 78.8% (171/217) of the women sampled were married (Table 1).

### 3.2. HPV Infection

Out of the 217 samples, 68 (31.3%) were positive for HPV DNA by PCR (Table 1). The prevalence of HPV infection was higher (50%) in women below 25 years and lower (23.9%) in women above 40 years. However, this difference was not statistically significant (*p* = 0.123). When HPV infection was assessed by marital status, single women had the highest rates of infection (*p* = 0.004). There was no association between HPV infection and level of education (*p* = 0.714), family planning (*p* = 0.072), and the number of children (*p* = 0.089).

### 3.3. HPV-HIV Coinfection

Of the 217 women, 29 (13.36%) were infected with HIV. The mean age for HIV-infected women was 40.7 years with a standard deviation of 6.6 and a median of 42 years. Of the 29 HIV-infected women, 16 (55.2%) were over 40 years. All the six women (100%) who were widowed or divorced were infected with HIV while 15/171 (8.8%) of the married women were infected with HIV. Age (*p* = 0.022) and marital status (*p* = 0.001) were seen to significantly influence HIV infection (Table 1).

The prevalence of coinfections of HPV and HIV was 6.5%. Fourteen of the 217 (6.5%) women studied had both HIV and HPV infection. HPV infections were significantly more prevalent in HIV-infected women (*p* = 0.031).

### 3.4. Cervical Cytology

**Figure 1:**
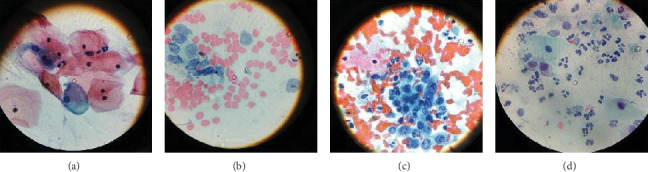
(a) Normal cells; (b) abnormal cytology results, a patient with adenocarcinoma; (c) abnormal cytology results, patient with HSIL; (d) patient with low-grade squamous intraepithelial cells.

Eight of the 217 (3.7%) women had abnormal smears (Table 1) with precancerous endpoints, 3/8 (37.5%) with HSIL (Figure 1(c)), 1/8 (12.5%) with LSIL (Figure 1(d)), and 3/8 (37.5%) with ASCUS, while one woman had adenocarcinoma cancer (Figure 1(b)). The highest rates of HPV infection was seen in women with abnormal cytology (*p* = 0.028). Six out of the eight women with abnormal cytology were HIV-HPV coinfected. There was 100% HPV infection in women with HSIL and cancer while only 1 out of 3 patients with ASCUS was HPV infected. A total of 102 women had normal cytology (Figure 1(a)). A further 107 (49.3%) had some form of inflammation due to various causes including candidiasis, cervicitis, bacterial vaginosis, and inflammation of unknown cause (Table 1). Of the 102 women with normal cytology, 32 (31.4%) were positive for HPV.

### 3.5. HPV Genotypes

Out of the 68 positive samples, only 60 were successfully identified as being HPV genotypes using BLAST (Table 2). The other eight sequences were too short (<100 bp) to cover the required LI region for genotyping of HPV and thus could not produce any similarity to known HPV. HPV 16 was the most prevalent (32.4%) with 22 samples being identified to have HPV 16 followed by HPV 6 (13.2%), HPV 81 (7.4%), HPV 73 (4.4%), HPV 52 (4.4%), HPV 35 (4.4%), HPV 33 (2.9%), HPV 26 (2.9%), HPV 66 (2.9%), HPV 83 (2.9%), HPV 31 (2.9%), HPV 102 (1.5%), HPV 11 (1.5%), HPV 51 (1.5%), HPV 58 (1.5%), and HPV 68 (1.5%).

### 3.6. HPV Genotypes and HIV Infection

A total of 7/29 (24.1%) HIV-infected women were infected with multiple high-risk HPV genotypes compared to 7/188 (3.7%) HIV-uninfected women (Table 3). Infections with multiple high-risk HPV types were greater in HIV-infected women than those in HIV-uninfected women with half of the coinfected women having multiple high-risk HPV infections (Table 4).

### 3.7. HPV Genotypes and Cytology

The patient who had presented with adenocarcinoma had multiple high-risk infections. This patient had HPV 35, 58, and 26 which are all high-risk types. HPV-infected women with normal cytology results had all risk types of HPV. A woman with HSIL had only low-risk HPV 81 (Table 5).

### 3.8. Phylogenetic Results

The sequences of samples with single HPV infections were used for phylogenetic analysis. These included HPV 6, 11, 16, 31, 33, 35, 51, 52, 58, 66, 81, and 83. Among all the HPV 16 samples, two variants were detected. HPV 81 and HPV 6 samples also had two variants each. A representative of each of the variants was used for the analysis.

**Figure 2:**
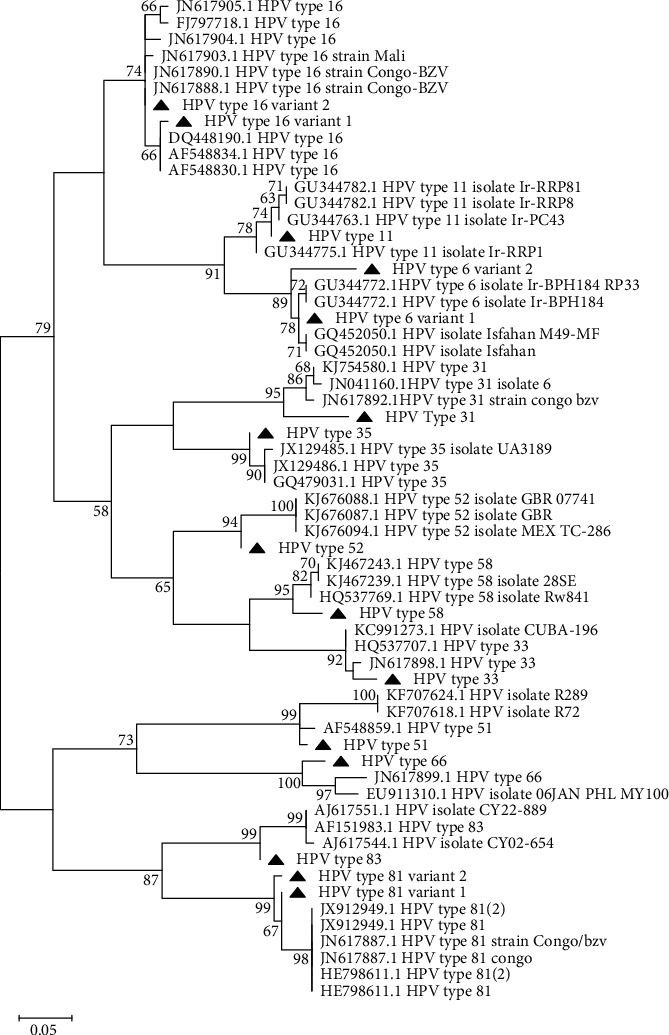
The evolutionary history was inferred by using the Maximum Likelihood method and the Tamura-Nei model [26]. The tree with the highest log likelihood (-1706.27) is shown. The tree is drawn to scale, with branch lengths measured in the number of substitutions per site.

Phylogenetic analysis was also able to cluster the HPV genotypes into high-risk types and low-risk types based on oncogenicity. Phylogeny clustered the sequences into two major groups. One that had HPV 51, 66, 81, and 83 and the second with the rest of the HPV types forming their own clade. This second clade was further clustered into three with HPV 6 and 11 forming a clade, HPV 16 variants forming another clade, and HPV 31, 35, 33, 52, and 58 forming a clade. Variant 1 of HPV 16 clustered with HPV 16 from Iran (accession numbers AF548830, AF548834, and DQ448190) while variant 2 clustered with HPV 16 from Africa specifically Mali and Congo (Figure 2).

## 4. Discussion

This study has described the HPV DNA prevalence of both HIV-infected and HIV-uninfected women attending the Kenyatta National Hospital Reproductive Health Clinic in Kenya. This clinic supports women for cervical cancer screening through pap test, family planning, fistula care, and STI clinic. Of these women, an overall HPV DNA prevalence of 31.3% is reported in this study. This is similar to the results of other studies in Kenya. Studies in Nairobi with 488 women and Tigoni with 438 women assessed HPV infection and cytology, and HIV infection revealed a HPV prevalence of 27% and 32.7%, respectively [7, 28]. This clearly indicates that HPV remains to be common in Kenya and the prevalence is still high. Eastern Africa has high incidence and mortality rates of cervical cancer which is the main cancer affecting women in this region with a risk of 1.5% for women below 65 years [29]. This high incidence and mortality as a consequence of HPV infection are due to high HIV burden and inadequate cervical cancer prevention services [3].

The prevalence of HPV was 28.7% in HIV-uninfected women and 48.3% in HIV-infected women. The high prevalence particularly for the HIV-uninfected women in this study may be due to high parity, increased number of sexual partners, and STI infection which are risky behavior for HPV acquisition [30]. This high prevalence of HPV in HIV-uninfected women was also recorded in a previous study done in Kenya and also in South African adolescent girls [7, 30]. However, in Nigeria, the prevalence of HPV in HIV-infected women was 54.1%. HIV-infected HPV-infected women tend to have aggressive forms of HPV due to their weakened immune system [6, 7]. A study in Uganda detected a very high HPV prevalence of more than 90% for any HPV genotype and 69% for high-risk HPV in women coinfected with HIV and HSV-2 [31].

The prevalence of HPV in this study in women with normal cytology was 31.3% which is slightly higher than 20.7% reported in other studies previously done in Kenya [28]. The prevalence of HPV in women with normal cytology is higher in sub-Saharan Africa than the rest of the world [3]. In this study, women with normal cytology had single and multiple high-risk HPV. A study in Morocco recorded a HPV prevalence of 34.3% in women with normal cytology [32] while HPV prevalence in the Kenyan population among the women who have normal cytology is 39.6% [33]. Even though cytology screening has dramatically reduced the mortalities associated with cervical cancer [34], it is prudent to include HPV DNA testing during routine diagnosis to cater to the women who have cytological normal results but may have high-risk HPV. In the present study, HPV infection was reported in 1/3 of the women with ASCUS cytology and 100% in HSIL and adenocarcinoma. This demonstrates the increasing prevalence of HPV with an increase in cervical intraepithelial lesions [33, 35]. A prevalence of 20.7% was noted for precancerous lesions in HIV-infected women in this study which is comparable to 25% reported in Nigeria [23].

HPV 16 was the most prevalent type in this study (32.4%). The high prevalence of HPV 16 has also been demonstrated in Kenya and worldwide [2, 3, 15, 36, 37]. A study done in Kenya revealed that HPV 16 and 68 were more prevalent in HIV-infected women than those without HIV [38]. In contrast, the second most prevalent high-risk type was HPV 81 (7.4%) followed by HPV 73, 35, and 52 each with a prevalence of 4.4%. This is in agreement with a meta-analysis indicating HPV 35 and 52 were the most prevalent in women with abnormal cytology [36, 37]. In this study, HPV 81 was the only genotype detected in a woman with HSIL. High prevalence of HPV 81 was also detected in women with abnormal cytology elsewhere [39, 40]. HPV 81 is recognized as a new type mostly found in patients with a weak immune system [40]. This type is associated with precancerous and cancerous lesions [39]. High HPV 35 prevalence was also recorded in Nigeria [23]. The high HPV prevalence of HPV 31 was recorded in HSIL and ASCUS in HIV-infected women in this study. This was also established in a study in Belgium among the HIV-infected women where HPV 31 has been found in a high number of HSIL cases [36, 37].

In the present study, one patient presented with adenocarcinoma after cytology examination. A cervical sample revealed multiple HPV infections of HPV 35, 58, and 26 which are all high-risk types. HPV 35 has been noted to be common in Africa compared to the rest of the world in women with adenocarcinoma [3]. Results elsewhere also indicate HPV 35 is associated with malignancies even in younger HIV-uninfected women [32]. In a study in Nigeria, HPV 35 was the most prevalent in HIV-infected women [23]. HPV 52 had a high prevalence in a study done in Western Kenya in HIV-infected women [41]. This high HPV 52 prevalence has also been observed in Zambia and China [42, 43]. A high prevalence of HPV 53 in a HIV-infected woman with invasive cancer in a study done in Mombasa Kenya also confirms the potential role of other high-risk genotypes in cervical cancer genesis especially in HIV-infected women [13, 14]. This is an indication that other HPV types are more prevalent in Kenya other than the two high-risk types included in the HPV vaccine.

The prevalence of high-risk HPV types in Kenya that are not included in the HPV vaccines available is a major concern, especially for HIV-infected women. This emphasizes the need for a regionally tailored vaccine in Kenya since other high-risk HPV types circulating in Kenya seem to play important roles in cervical cancer etiology. A meta-analysis study in Kenya showed that in women with abnormal cytology high-risk HPV 16, 35, and 52 are prominent and HPV 16/18 is common in women with invasive cervical cancer [36, 37]. Another study on adolescent girls in South Africa demonstrated a high prevalence of nonvaccine high-risk HPV types in these girls and concluded that the bivalent vaccine available would not be effective in preventing infections due to the high-risk HPV in that region [8]. Zhang et al., [43] recommended that HPV 16, 58, 33, and 52 testing should be included during routine cytology examination due to their prevalence in Shenzen, China. This shows that HPV genotypes seem to vary by region. In addition, vaccinated women will still need screening for other high-risk types [36, 37]. Furthermore, cross-reactivity of antibodies is within species and not across species. Thus, antibodies against HPV 18 may cross-react with HPV 45 because they belong to the same species (Species 7) but not with HPV 16, which is from a different species (Species 9) [16]. Likewise, antibodies against HPV 16 will cross-react with HPV 33, 35, 52, and 58 from the same species (species 9) but not with HPV 18 and 45. However, Menon et al., suggest that this cross-reactivity is not achieved with a single-dose vaccination schedule [14]. Other studies seem to differ on this showing that Cervarix vaccine offers cross-protection to HPV 31, 33, and 45 [11]. A 9 valent vaccine with HPV 16, 18, 11, 6, 31, 33, 45, 52, and 58 is estimated to prevent up to 90% of all cancers [12–14]. However, this vaccine is expensive and does not prevent infection beyond the 9 HPV types in the vaccine but is still a better alternative for sub-Saharan Africa [12]. It is also a preferred alternative for China [43, 44]. In Kenya, HPV vaccination was rolled out in 2019 where girls aged 9 years are given the bivalent vaccine Gardasil. Of great concern is the lack of data on HPV vaccination effectiveness in HIV-infected women due to the lower immune responses in these women [45].

The phylogenetic analysis was able to cluster the HPV types in the current study based on the genotypes in addition to their oncogenic potential and pathogenicity. Genotypes that group together share common carcinogenicity and the kind of host tissues they infect [46]. Cutaneous HPVs rarely infect the mucosa [16]. The HPV 16 phylogenetic tree depicts the origin of the isolates and groups them by their geography as European, African 1, African 2, and Asian American [18]. In this study, HPV 16, 6, and 81 had two variants each. There is a strong relationship between other HPV 16 types other than the European isolates with the severity of cytological lesions and even cancer [18]. This suggests that the majority of African HPV 16 circulating in Kenya might be very virulent. HPV variants are thought to have diverged through genetic drift which is evidenced by the grouping of HPV 16 based on geography or human ethnic group [46]. HPV 31, 33, 35, 52, and 58 which formed a distinct cluster in this study are HPV 16-related alpha 9 variants that are the most oncogenic causing 75% of cancers worldwide [47]. HPV 83 and 81 that clustered together are low-risk HPVs belonging to alpha-3 species [9].

## 5. Conclusion

The present study established a HPV prevalence of 31.3% and HPV-HIV coinfection of 6.5% among HIV-infected and HIV-uninfected women attending the Kenyatta National Hospital Reproductive Health Clinic in Kenya. This study showed that HPV 16 is the most prevalent HPV genotype in these women. This study also revealed the circulation of other HPV types: HPV 81, 35, 31, 51, 45, 73, 58, and 26 in the Kenyan population which play an important role in cancer etiology but are not included in the HPV vaccine. This study recommends fast-tracking HPV screening especially of HIV-infected women and those women with abnormal cytology. There is need to improve the current vaccine by including the other high-risk types prevalent in this region.

## Figures and Tables

**Table 1:** Demographic characteristics, HPV, and HIV Infections among women attending the Reproductive Health Clinic at a referral hospital in Nairobi, Kenya, in 2013 and 2014.

	Number *N* = 217	HPV			HIV		
		HPV positive	HPV negative	*p* value	HIV infected	HIV uninfected	*p* value
Age							
<25	14 (6.5)	7 (50.0)	7 (50.0)	0.123	1 (7.1)	13 (92.9)	0.022^∗^
25-39	132 (60.8)	44 (33.3)	88 (66.7)		12 (9.1)	120 (90.9)	
Over 40	71 (32.7)	17 (23.9)	54 (76.1)		16 (22.5)	55 (77.5)	
Marital status							
Singles	40 (18.4)	21 (52.5)	19 (47.5)	0.004^∗^	8 (20.0)	32 (80.0)	0.001^∗^
Married	171 (78.8)	45 (26.3)	126 (73.7)		15 (8.8)	156 (91.2)	
Divorced or widowed	6 (2.8)	2 (33.3)	4 (66.7)		6 (100)	0 (0.0)	
Level of education							
None	2 (9)	1 (1.5)	1 (0.7)	0.714	1 (50)	1 (50.0)	0.073
Primary	54 (29.9)	15 (22.1)	39 (26.2)		10 (18.5)	44 (81.5)	
Secondary	97 (44.7)	33 (48.5)	64 (43.0)		14 (14.4)	83 (85.6)	
Tertiary	64 (29.5)	19 (27.9)	45 (30.2)		4 (6.3)	60 (93.8)	
Number of live children							
None	18 (8.3)	10 (14.7)	8 (5.4)	0.096	3 (16.7)	15 (83.3)	0.959
One	35 (16.1)	12 (17.6)	23 (15.4)		4 (11.4)	31 (88.6)	
Two	77 (35.5)	24 (35.3)	53 (35.6)		10 (13.0)	67 (87.0)	
Three or more	87 (40.1)	22 (32.4)	65 (43.6)		12 (13.8)	75 (86.2)	
Family planning method							
Hormonal	50 (23.0)	11 (22.0)	39 (78.0)	0.120	2 (3.8)	51 (96.2)	0.031^∗^
Nonhormonal	167 (77.0)	57 (34.1)	110 (65.9)		27 (16.5)	137 (83.5)	
Cytology results							
Normal	102 (47.0)	32 (31.4)	70 (68.6)	0.028^∗^	9 (31.0)	93 (49.5)	0.001^∗^
Abnormal	8 (3.7)	6 (75.0)	2 (25.0)		6 (20.7)	2 (1.1)	
Inflammation	107 (49.3)	30 (28.0)	77 (72.0)		14 (48.3)	93 (49.5)	
HIV status							
Infected	29 (13.4)	14 (48.3)	15 (51.7)	0.031^∗^			
Uninfected	188 (86.6)	54 (28.7)	134 (71.3)				

^∗^ *p* value is significant; percentages are in brackets.

**Table 2:** HPV genotypes identified as single or multiple infections in women attending a Reproductive Health Clinic in Kenya in 2013 and 2014.

Type of infection	HPV genotypes	Number *N*
Single high-risk infections	16	6
	52	3
	31	1
	58	2
	35	2
	33	2
	73	2
	51	1
	66	1

Single low-risk infections	81	2
	6	8
	11	1

Multiple infections with high risk	16, 31	9
	16, 31, 33	2
	35, 58, 26	1
	73, 68, 45	1
	33, 35	1

Multiple infections with high- and low-risk HPV	81, 51	2
	26, 6, 16, 31, 35	1
	81, 16	2
	16, 11	1
	11, 87, 45, 16	1
	66, 11, 45	1
	26, 51, 16, 35, 6	1
	83, 16	1
	83, 31, 16	1
	102, 73	1
	83, 73	2

Multiple low-risk infections	102, 83	1
Unidentified		8
Total		68

**Table 3:** HPV genotype distribution and HIV infection in women attending a Reproductive Health Clinic in Kenya in 2013 and 2014.

HPV genotype	HIV infected *N* = 29 (%)	HIV uninfected *N* = 188 (%)
Single infections with high-risk types	3 (10.3)	16 (8.5)
Single low-risk infections	1 (3.4)	10 (5.3)
Multiple high-risk infections	7 (24.1)	7 (3.7)
Multiple high- and low-risk infections	3 (10.3)	8 (4.3)

**Table 4:** HPV genotype distribution among the fourteen HPV- and HIV-coinfected women attending a Reproductive Health Clinic in Kenya in 2013 and 2014.

Risk	Genotypes in HPV and HIV coinfected
Single infections with high-risk types	16 (2)
	51

Single low-risk infections	81

Multiple high-risk infections	35, 58, 26
	16, 31
	73, 68, 45
	16, 31 (2)
	33, 35
	16, 31, 33

Multiple high- and low-risk infections	81, 16
	83, 73
	16, 11, 87, 45

**Table 5:** HPV genotypes with cytology results in women attending a Reproductive Health Clinic in Kenya in 2013 and 2014.

Risk	Genotypes				
	Normal	Inflammation	Cancer	HSIL	ASCUS
Single high-risk infections	51	16			
	16	52			
	35	31			
	73	33			
	58	73			

Multiple high risk	16, 31	16, 31	35, 58, 26	16, 31	16, 33, 31
	16, 31, 33	33, 35		73, 68, 45	16, 31

Multiple low and high	81, 51	16, 11			
	81, 16	16, 11, 87, 45			
	26, 6, 35, 16, 31	83, 16			
	66, 11, 45	83, 31, 16			
	26, 51, 16, 35	83, 73			
		102, 73			

Single low risk	6	6		81	
		81			
		11			

Multiple low risk	102, 83				

## Data Availability

The HPV sequence data used to support the findings of this study have been deposited at the DNA Data Bank of Japan (DDBJ) accession numbers LC155219-LC155254. Personal data of the patients including filled questionnaires are restricted by the Ethical Board (Kenya Medical Research Institute Scientific and Ethics Review and the Kenyatta National Hospital Ethics Review Committee) in order to protect patient privacy.

## References

[1] Bouvard V., Baan R., Straif K. (2009). A review of human carcinogens--part B: biological agents. *Lancet Oncology*.

[2] Clifford G. M., Smith J. S., Aguado T., Franceschi S. (2003). Comparison of HPV type distribution in high-grade cervical lesions and cervical cancer: a meta-analysis. *British Journal of Cancer*.

[3] De Vuyst H., Alemany L., Lacey C. (2013). The Burden of Human Papillomavirus Infections and Related Diseases in Sub- Saharan Africa. *Vaccine*.

[4] WHO (2010). *Cancer of the cervix in the African region: current situation and way forward*.

[5] Munoz M., Camargo M., Soto-de Leon S. C. (2013). Human papillomavirus detection from human immunodeficiency virus-infected Colombian Women's paired urine and cervical samples. *PLoS One*.

[6] Strickler H. D., Burk R. D., Fazzari M. (2005). Natural history and possible reactivation of human papillomavirus in human immunodeficiency virus–positive women. *Journal of the National Cancer Institute*.

[7] Yamada R., Sasagawa T., Kirumbi L. W. (2008). Human papillomavirus infection and cervical abnormalities in Nairobi, Kenya, an area with a high prevalence of human immunodeficiency virus infection. *Journal of Medical Virology*.

[8] Adler D. H., Wallace M., Bennie T. (2014). Cervical Dysplasia and High-Risk Human Papillomavirus Infections among HIV-Infected and HIV-Uninfected Adolescent Females in South Africa. *Infectious Diseases in Obstetrics and Gynaecology*.

[9] Schiffman M., Herrero R., DeSalle R. (2005). The carcinogenicity of human papillomavirus types reflects viral evolution. *Virology*.

[10] Villa L. L., Costa R. L. R., Petta C. A. (2005). Prophylactic quadrivalent human papillomavirus (types 6, 11, 16, and 18) L1 virus-like particle vaccine in young women: a randomised double-blind placebo- controlled multicentre phase II efficacy trial. *Lancet Oncology*.

[11] Harper D. M., Vierthaler S. L. (2011). Next Generation Cancer Protection: The Bivalent HPV Vaccine for Females. *ISRN Obstetrics and Gynecology*.

[12] Joura E. A., Giuliano A. R., Iversen O. E. (2015). A 9-valent HPV vaccine against infection and intraepithelial neoplasia in women. *New England Journal of Medicine*.

[13] Menon S., Luchters S., Rossi R. (2018). Human papilloma virus correlates of high grade cervical dysplasia in HIV-infected women in Mombasa, Kenya: a cross-sectional analysis. *Virology Journal*.

[14] Menon S., Rossi R., Kariisa M., Callens S. (2018). Determining the HPV vaccine schedule for a HIV-infected population in sub Saharan Africa, a commentary. *Virology Journal*.

[15] Ngugi C. W., Schmidt D., Wanyoro R. K., Boga H., Wanzala P., Muigai A. W. T. (2011). Prevalence of human papillomavirus infection by age and cervical cytology in Thika, Kenya. *African Journal of Health Sciences*.

[16] de Villiers E.-M., Fauquet C., Broker T. R., Bernard H.-U., Hausen H. z. (2004). Classification of papillomaviruses. *Virology*.

[17] Lam T. T.-Y., Hon C.-C., Tang J. W. (2010). Use of phylogenetics in the molecular epidemiology and evolutionary studies of viral infections. *Critical Reviews in Clinical Laboratory Science*.

[18] Ntova C. K., Kottaridi C., Chranioti A. (2012). Genetic variability and phylogeny of high risk HPV type 16, 18, 31, 33 and 45 L1 gene in Greek women. *Internatinal Journal of Molecular Sciences*.

[19] Pourhoseingholi M. A., Vahedi M., Rahimzadeh M. (2013). Sample size calculation in medical studies. *Gastroenterology and Hepatology from Bed to Bench.*.

[20] Husain O. A. N., Butler E. B., Evans D. M. D., Macgregor J. E., Yule R. (1974). Quality control in cervical cytology. *Journal of Clinical Pathology*.

[21] Noreh J., Sekadde-Kigondu C., Karanja J. G., Thagana N. G. (1997). Median age at menopause in a rural population of western Kenya. *East African Medical Journal*.

[22] NASCOP (2009). *National Guidelines for HIV Testing and Counselling in Kenya*.

[23] Maimako Y. M., Fowotade A., Anaedobe C. G., Manga M. M., Bakare R. A., Abimiku B. A. (2019). Human papillomavirus correlates of high grade cervical dysplasia among HIV-infected women at a major treatment centre in Nigeria: a cross-sectional study. *Pan African Medical Journal*.

[24] Nayar R., Wilbur D. C. (2015). *The Bethesda System for Reporting Cervical Cytology*.

[25] Kumar S., Stecher G., Li M., Knyaz C., Tamura K. (2018). MEGA X: molecular evolutionary genetics analysis across computing platforms. *Molecular Biology and Evolution*.

[26] Tamura K., Nei M. (1993). Estimation of the number of nucleotide substitutions in the control region of mitochondrial DNA in humans and chimpanzees. *Molecular Biology and Evolution*.

[27] Page R. D. M. (1996). Tree view : an application to display phylogenetic trees on personal computers. *Cabios Application Note*.

[28] Muchiri L., Sekkade-Kigondu C. B., Ndirangu G. (2012). The impact of human immunodeficiency virus and human papillomavirus co-infection on HPV genotype distribution and cervical lesion grade in a semi-urban population in Tigoni, Kenya. *African Journal of Pharmacology and Therapeutics*.

[29] Parkin D. M., Bray F., Ferlay J., Pisani P. (2005). Global cancer statistics, 2002. *CA: A Cancer Journal for Clinicians*.

[30] Mbulawa Z. Z. A., van Schalkwyk C., Hu N. C. (2018). High human papillomavirus (HPV) prevalence in south African adolescents and young women encourages expanded HPV vaccination campaigns. *PLoS One*.

[31] Rositch A. F., Gravitt P. E., Tobian A. A. R. (2013). Frequent detection of HPV before and after initiation of antiretroviral therapy among HIV/HSV-2 co-infected women in Uganda. *PLoS One*.

[32] Souho T., el Fatemi H., Karim S. (2016). Distribution of carcinogenic human papillomavirus genotypes and association to cervical lesions among women in fez (Morocco). *PLoS One*.

[33] Bruni L., Barriotevo-Rosa L., Serrano B. (2014). *Human papillomavirus and related diseases in Kenya*.

[34] Persson M., Elfström K. M., Olsson S.-E., Dillner J., Andersson S. (2015). Minor cytological abnormalities and up to 7-year risk for subsequent high-grade lesions by HPV type. *PLoS One*.

[35] ICO HPV Information Centre (2016). *Human papillomavirus and related diseases report*.

[36] Menon S., Rossi R., Benoy I., Bogers J. P., van den Broeck D. (2018). Human papilloma virus infection in HIV-infected women in Belgium : implications for prophylactic vaccines within this subpopulation. *European Journal of Cancer Prevention*.

[37] Menon S., Wusiman A., Boily M. C. (2016). Epidemiology of HPV genotypes among HIV positive women in Kenya : a systematic review and meta-analysis. *PLoS One*.

[38] Ermel A., Tonui P., Titus M. (2019). A cross-sectional analysis of factors associated with detection of oncogenic human papillomavirus in human immunodeficiency virus-infected and uninfected Kenyan women. *BMC Infectious Diseases*.

[39] Choi Y.-D., Han C. W., Chung W. J. (2009). Analysis of HPV-other samples by performing HPV DNA sequencing. *The Korean Journal of Pathology*.

[40] Co N. N. C., Chu L.-O., Chow J. K. F., Tam J. W. O., Ng E. K. O. (2013). HPV prevalence and detection of rare HPV genotypes in Hong Kong women from southern China with cytological abnormalities. *ISRN Virology*.

[41] Dainty E. E. (2013). *Screening for cervical cancer in HIV positive Kenyan women: the role of HPV*.

[42] Sahasrabuddhe V. V., Mwanahamuntu M. H., Vermund S. H. (2007). Prevalence and distribution of HPV genotypes among HIV-infected women in Zambia. *British Journal of Cancer*.

[43] Zhang Y., Wang Y., Liu L., Guo C., Liu Z., Nie S. (2016). Prevalence of human papillomavirus infection and genotyping for population-based cervical screening in developed regions in China. *Oncotarget*.

[44] Li Z., Liu F., Cheng S. (2016). Prevalence of HPV infection among 28,457 Chinese women in Yunnan Province, southwest China. *Scientific Reports*.

[45] Lacey C. J. N. (2019). HPV vaccination in HIV infection. *Papillomavirus Research*.

[46] Burk R. D., Chen Z., van Doorslaer K. (2009). Human papillomaviruses : genetic basis of carcinogenicity. *Public Health Genomics*.

[47] Chen Z., Schiffman M., Herrero R. (2011). Evolution and taxonomic classification of human papillomavirus 16 (HPV16) -related variant genomes : HPV 31, HPV 33, HPV35, HPV 52, HPV58 and HPV 67. *PLoS One*.

